# Cutaneous Permeation of a Percutaneously Applied Glucocorticoid Using Plant-Based Anionic Phospholipids in Hydrogenated Vegetable Oil: A Preliminary Study

**DOI:** 10.3390/medicina58101334

**Published:** 2022-09-23

**Authors:** Jack V. Greiner, Hridaya N. Bhargava, Thomas Glonek, Donald R. Korb, Michael E. Lindsay, Paula J. Oliver

**Affiliations:** 1 Department of Ophthalmology, Harvard Medical School, 20 Staniford St., Boston, MA 02114, USA; 2 Schepens Eye Research Institute of Massachusetts Eye and Ear Infirmary, Boston, MA 02114, USA; 3 Clinical Eye Research of Boston, 5 Whittier Place, Ste 102, Boston, MA 02114, USA; 4 Division of Pharmaceutical Sciences, Massachusetts College of Pharmacy and Health Sciences University, Boston, MA 02115, USA; 5 Ocular Research of Boston, Inc., Boston, MA 02116, USA

**Keywords:** anionic polar phospholipids (phosphatidylglycerol, phosphatidylinositol, phosphatidylserine), corticosteroids, skin, skin cream

## Abstract

*Purpose*: It is important that, when corticosteroids are used therapeutically, concentrations be reduced as much as possible to mitigate potential adverse events and side effects. This preliminary study compares the permeation for the delivery of a corticosteroid in a 1% hydrocortisone-supplemented topical cream containing anionic polar phospholipids (APP) in hydrogenated vegetable oil (triglyceride) versus a market-leading 1% hydrocortisone in a mineral hydrocarbon-based skin cream. *Methods*: Using the Franz diffusion cell method with cadaveric skin, the permeation of a 1% hydrocortisone-supplemented cream containing APP (test preparation) was compared with a commercially available 1% hydrocortisone cream (control preparation). The principal APP in the test preparation were phosphatidylinositol, phosphatidylserine and phosphatidylglycerol. Permeation was determined at 4 and 8 h time intervals. *Results*: The permeation values for the 1% hydrocortisone supplemental APP cream (test preparation) were comparatively very high 1180 ng/cm^2^ at 4 h and 2173 ng/cm^2^ at 8 h, in contrast to the 1% hydrocortisone cream (control preparation) values of 13 ng/cm^2^ at 4 h and 98 ng/cm^2^ at 8 h. Permeation of skin cream increased significantly from 0 to 4 and 8 h, when comparing the APP test preparation with the control preparation (*p* < 0.001). This translates, respectively, into the 90-fold greater and a 20-fold greater penetration of the test preparation APP cream over the 1% hydrocortisone cream at 4 h and 8 h time points. *Conclusions*: This preliminary study demonstrates the enhanced permeation of 1% hydrocortisone when applied topically to the skin in an APP skin cream vehicle. This enhanced permeation suggests the potential use of APP technology to deliver therapeutically effective hydrocortisone treatment to the skin at markedly reduced concentrations of steroid. As such, APP technology may offer an improved approach to the treatment of dermatoses associated with inflammatory diseases and conditions requiring prolonged topical corticosteroid therapy.

## 1. Introduction

Inflammatory dermatoses are prevalent and are most often accompanied by desiccation or dryness of the skin. Effective pharmaceutical treatments of these maladies are limited. The most common treatment is the use of topical corticosteroids. The use of corticosteroids on skin is problematic as they are known to have an exhaustive list of adverse effects [1,2]. This preliminary study reports on the discovery of a topical delivery system using anionic polar phospholipid (APP) technology [3,4] that is capable of providing therapeutic concentrations of corticosteroid to the skin with markedly increased permeation rates. This delivery system using APP technology suggests the potential for enhanced topical drug delivery at lower concentrations and doses of corticosteroids.

Topically applied skin creams with anionic phospholipids (APP) have demonstrated rapid healing of inflammatory skin conditions caused by desiccation of the skin (e.g., dryness, eczema, psoriasis, and diabetes mellitus) [3,4]. A phase III trial conducted at St. Jude Children’s Research Hospital (Memphis, TN, USA) compared APP skin cream with an aloe-vera-based gel in the prevention and treatment of dermatoses secondary to radiation burns [5,6]. This phase III trial demonstrated rapid healing with less discomfort using the APP skin cream employing APP technology. The large increase in permeation of APP skin cream is believed to be the result of the negatively charged amphipathic lipid ingredients. The APP are water-seeking, enabling permeation of the outmost layer of the skin, the stratum corneum [7]. APP molecules self-organize in the presence of water and are attracted to the water layers within the stratum corneum and the deeper skin layers [7]. The increased permeation of skin creams with negatively charged phospholipids offers significant potential for use as a vehicle for improved drug delivery [3,4].

APP molecules are bio-chemicals that are present naturally in the body. For example, APP molecules are found normally in the meibomian glands of the eyelids [8,9]. They also are natural components of the skin of the eyelid [10] and animal cells. APP technology has been used successfully in treating dry eye [11], producing a very stable tear film lipid layer (outermost layer of the tear film), the importance of which has been demonstrated [12]. The success of APP technology in the treatment of conditions of the ocular surface presents the potential for the use of APP technology to be expanded for treatments of other conditions, such as those of the skin [3,4].

Use of currently available topical corticosteroids is associated with adverse events, such as corticosteroid-induced dermal atrophy, which can be uncomfortable, cause an undesired cosmetic appearance, and opens pathways for infection due to the side effect of skin thinning [2]. This detrimental response is especially significant in regions of exceptionally thin skin. The skin of the eyelid, for example, is the thinnest skin in the body and, therefore, susceptible to this type of damage. Steroid treatment can result in thinning to such a severe extent that normally smooth skin can deteriorate and develop the crumpled appearance of crepe paper.

This preliminary study reports the increased permeation and delivery of corticosteroids to the skin through use of a topical cream containing APP. These findings support the possibility of delivering effective therapy at markedly reduced corticosteroid concentrations, which might decrease the incidence of adverse events and side effects related to corticosteroid use.

## 2. Methods

**Figure 1:**
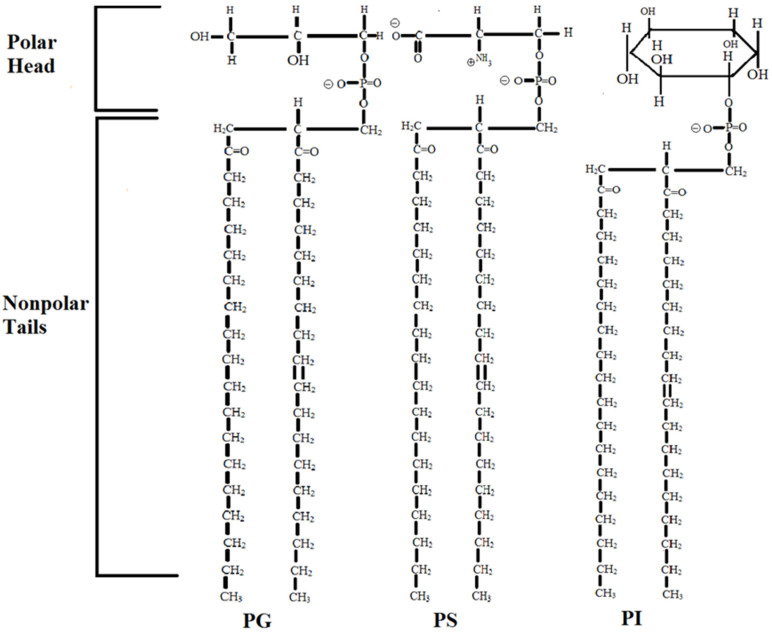
Diagrams of the chemical structure of the anionic polar phospholipids phosphatidylglycerol (PG), phosphatidylserine (PS), and phosphatidylinositol (PI). These 3 phospholipids are extremely important in binding water and creating extended hydration-layer structures.

In this preliminary study, the APP-facilitated permeation of hydrocortisone was studied through cadaver skin permeation studies utilizing the Franz diffusion cell method [13]. No ethics committee approval was required for the conduct of this study because none of the ingredients in the skin cream emulsions were derived from living human or animal tissues, fluids, or cell lines. Briefly, the Franz diffusion cell method requires that the apparatus be set up in the typical method with two adjacent chambers, a donor chamber and a receptor chamber, separated by an intact adult cadaver skin membrane. The donor compartment contained the test product—a 1% hydrocortisone-supplemented APP skin cream. The APP component principally contained the phospholipids phosphatidylglycerol, phosphatidylserine, and phosphatidylinositol (Figure 1) (in which their concentration more preferably varied between 1.0 and 25 percent by weight of the composition). Additional components of the test product included isopropyl palmitate, cetyl alcohol, stearyl alcohol, beeswax, stearic acid, glycerol monostearate, sodium hydroxide, myristyl myristate, propylene glycol, benzyl alcohol, methyl-propyl paraben, Carbopol, oleic acid, Arlacel-60, Tween-60, water, and hydrogenated triglycerides [4]. A commercially available 1% hydrocortisone cream (Hytone ^®^ cream (Dermik Laboratories, Bridgewater, NJ, USA)) was selected as a control because this product represents a market leader in the effective treatment of inflammatory conditions of the skin. The receptor compartment was filled with phosphate-buffered saline (PBS). Diffusion of the drug from the test product across the cadaver skin membrane was monitored by assay of sequentially collected samples of the receptor medium. Assays using the test product as well as the control product were each repeated in quadruplicate. In the present study, aliquots (*n* = 5) of receptor medium were removed at 4 h and 8 h time points and analyzed by high-pressure liquid chromatography; permeation of hydrocortisone was determined in nanograms/cm^2^. Since this was a preliminary study, permeation was measured at only two time points; time points were selected to coincide with those typically used for skin treatment clinically.

## 3. Results

**Figure 2:**
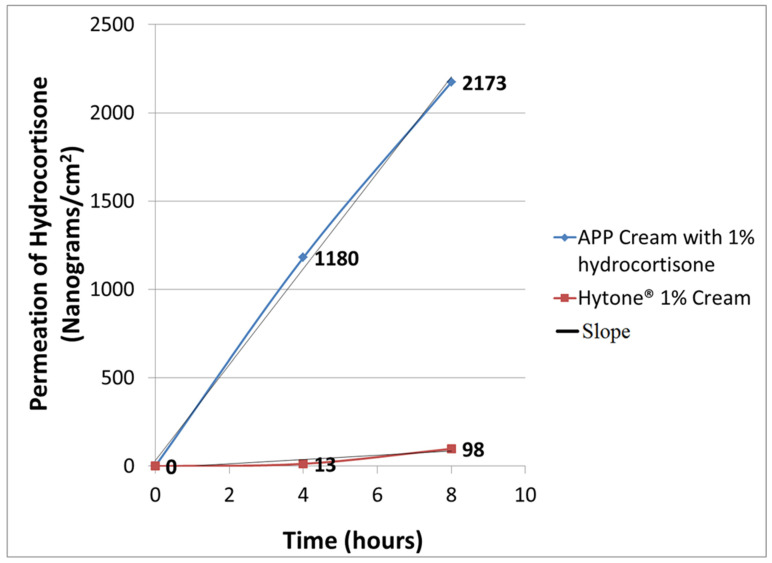
Permeation of 1% hydrocortisone steroid in APP plant-based cream (control product) vs. Hytone^®^ 1% mineral hydrocarbon-based cream (control product). Assuming a linear relationship, the slope of APP skin cream with 1% hydrocortisone y = 271.63 x + 31.67 and the slope of Hytone^®^ 1% cream y = 12.25 x − 12. The variability at the 4 h and 8 h time points among quadruplicate samples in test and control products was too small to be plotted in the figure.

Hydrocortisone permeation with the control cream was 13 ± 0.5 ng/cm^2^ at 4 h and 98 ±2 ng/cm^2^ at 8 h, whereas the values for the corticosteroid-supplemented APP skin cream were 1180 ±27 ng/cm^2^ at 4 h and 2173 ±45 ng/cm^2^ at 8 h (Figure 2). Results from repeated measurements in both the control hydrocortisone cream and the corticosteroid-supplemented AAP skin cream showed a less than 5% difference at both 4 h and 8 h time points. This translates, respectively, into 90-fold greater and 20-fold greater steroid penetration using the hydrocortisone-supplemented APP cream over the control cream at the 4 h and 8 h time points. The results suggest that the use of the corticosteroid-supplemented APP skin cream might permit the effective delivery of steroid at a rate of penetration up to two orders of magnitude greater than the compared control cream, a 1% hydrocortisone skin cream without APP.

## 4. Discussion

This preliminary study demonstrates the enhanced permeation of hydrocortisone using the Franz diffusion cell method [13] when tested in a 1% hydrocortisone-supplemented plant-based APP-supplemented triglyceride-based skin cream vehicle. With these findings, our study suggests the potential of APP technology for enhanced hydrocortisone delivery to the skin at markedly reduced steroid doses. The unique biochemical and biophysical properties of the APP vehicle (test preparation) provide better hydration of skin tissue [7,14], as well as an improved mechanism for transporting the corticosteroid, both of which ultimately lead to enhanced skin permeation. These properties are particular to the APP test preparation and are not a component of the control preparation, suggesting their role in the increased permeation of the APP skin cream.

Reportedly, hydrating a tissue, such as skin, can enhance the permeability of the tissue and, thus, lead to the increased permeation of a pharmaceutical [15]. APP may improve the hydration of skin tissue via two mechanisms. First, dry skin hydration can be accomplished via repair of the skin’s lamellar system [16]. APP skin cream has a unique structure that forms a bilayer component in contact with water, in which one or more polar terminus groups and one or more non-polar terminus groups of the phospholipids (Figure 1) form a bilayer. The polar and non-polar groups are separated from each other by a spacer segment, the esterified glycerol backbone residue of the phospholipid molecule [3,4]. The bilayer forms an organized, aligned lamellar structure within the epidermis, most likely the stratum corneum, where adjacent bilayers are separated by a water layer [17].

The APP provide a process to repair defects (holes) in the lamellar strata that result from skin damage and concurrent loss of the natural polar lipid components, which can result in dehydration. This repair replenishes the bilayer lamellae, which naturally occur in healthy skin. The APP vehicle also contains neutral lipids (triglycerides). When a neutral lipid is contained within the treatment composition, the neutral lipid forms an aligned layer between the non-polar groups of the bilayer, thus providing greater lubricity between corneocytes [3,4]. Therefore, by assisting in the prevention of transepidermal water loss from the skin, APP contribute to the hydration of skin tissue and, thus, increase the permeation of pharmaceuticals.

In addition to preventing transepidermal water loss through repair, APP skin cream technology is able to increase hydration via a second mechanism: the supplementation of the glycoprotein and glycolipid humectants within the strata of the epithelium by the hydroxylated phospholipids of the APP component. Because phospholipids and glycerides possess polar head groups, they seek water, thus creating a driving force causing the phospholipids and glycerides to penetrate the epidermis and form a bilayer structure within the stratum corneum [3,4]. It is presumed that the glycoproteins occupy hydrophilic spaces among the corneocytes of the stratum corneum. The starch-like portions of these molecules are highly hygroscopic and, as such, these APP molecules attract and organize water molecules, leading to increased hydration of tissue.

With this increased hydration, the improved penetration of steroids by APP skin cream is also a result of APPs’ enhanced ability to transport therapeutic agents known to be insoluble in water—in particular, steroids. As has been discussed above, APP repair damaged lamellae and create bilayers similar to those found in healthy skin tissue and are characteristic of animal cell membranes. Cell membranes mostly are composed of phospholipids arranged with their hydrophilic head groups facing the aqueous medium and their fatty acyl tails forming an underlying hydrophobic membrane layer [18]. As steroids are insoluble in water, lipoprotein carriers, such as high-density lipoprotein (HDL), are likely to be important in the transport and metabolism of steroids, such as hydrocortisone, in human plasma [19]. The structure of HDL, which may be viewed as a well-characterized portion of a phospholipid membrane, consists of a polar monolayer formed around a hydrophobic core with cholesterol molecules intercalated between the phospholipids [20]. Hydrophobic compounds, such as triglycerides, do not integrate into the phospholipid membrane, but, rather, are positioned beneath the hydrophobic side chain tails of the phospholipids, either in a hydrophobic core, as in HDL [20], or, in the case of skin, between the two planes of the phospholipid membrane lamellar bilayers. In APP skin cream, the oil component is hydrophobic and is integrated into the hydrophobic region between phospholipid membranes, repairing damaged holes in the lamellae structure [17]. The APP molecules, phosphatidylinositol (PI), phosphatidylserine (PS), and phosphatidylglycerol (PG), are believed to be the same negatively charged phospholipids that are present in HDL [21]. These molecules are incorporated into the skin epidermal membranes along with steroid molecules, such as cholesterol, to restore epidermal bilayers.

Although this study is preliminary, it presents evidence in support of the above findings, and, further, elucidates the possible benefit of APP skin cream to deliver doses of corticosteroid effectively. APP appears to have an enhanced ability to penetrate and hydrate skin [7] in conjunction with an apparent capability to intercalate corticosteroids into the phospholipid membrane in the same manner as HDL. APP cream not only demonstrates increased repairing effects [7,14], but also may function as an excellent vehicle for the pharmaceutical delivery of steroids deep into the lamellar structure of the skin.

## 5. Conclusions

Whereas the control mineral hydrocarbon-based steroid cream permeates the skin to a limited degree, the steroid-supplemented APP plant-based skin cream in this preliminary study exhibits a high degree of permeability. As such, the use of APP skin cream in the management of skin conditions offers the potential for a reduction in the concentration of steroids prescribed.

## Data Availability

The data are presented in the text.

## References

[1] Hengge U.R., Ruzicka T., Schwartz R.A., Cork M.J. (2006). Adverse effects of topical glucocorticosteroids. J. Am. Acad. Dermatol..

[2] (2016). Hydrocortisone. http://www.pdr.net.

[3] Korb D.R., Glonek T., Greiner J.V. (1998). Skin Cream Preparation and Method. U.S. Patent.

[4] Korb D.R., Glonek T., Greiner J.V. (1998). Skin Care Preparation and Method. U.S. Patent.

[5] Bosley C., Smith J., Baratti P., Pritchard D.L., Xiong X., Li C., Merchant T.E. (2003). A phrase III trial comparing an anionic phospholipid-based (APP) cream and aloe vera-based gel in the prevention and treatment of radiation dermatitis. Int. J. Radiat. Oncol. Biol. Phys..

[6] Merchant T.E., Bosley C., Smith J., Baratti P., Pritchard D., Davis T., Li C., Xiong X. (2007). A phase III trial comparing an anionic phospholipid-based (APP) cream and aloe vera-based gel in the prevention and treatment of radiation dermatitis in pediatric patients. Radiat. Oncol..

[7] Glonek T., Greiner J.V., Oliver P.J., Baker T.L. (2022). Implications of a diabetic foot xerosis treatment with an emulsion containing the plant-based anionic phospholipoids. J. Prim. Care Community Health.

[8] Greiner J.V., Glonek T., Korb D.R., Booth R., Leahy C.D. (1996). Phospholipids in Meibomian gland secretion. Ophthalmic Res..

[9] Greiner J.V., Glonek T., Korb D.R., Leahy C.D. (1996). Meibomian gland phospholipids. Curr. Eye Res..

[10] Greiner J.V., Glonek T., Townsend D.J., Hearn S.L. (1998). Epidermal and dermal phospholipids of the human eyelid. A ^31^P nuclear magnetic resonance spectroscopy study. Arch. Dermatol. Res..

[11] Korb D.R., Greiner J.V., Glonek T. (2002). The effects of anionic and zwitterionic phospholipids on the tear film lipid layer. Adv. Exp. Med. Biol..

[12] Korb D.R., Baron D.F., Herman J.P., Finnemore V.M., Exford J.M., Hermosa J.L., Leahy C.D., Glonek T., Greiner J.V. (1994). Tear film lipid layer thickness as a function of blinking. Cornea.

[13] Franz T., Lehman P., Swarbrick J., Boyland J. (1995). Percutaneous absorption. Encyclopedia of Pharmaceutical Technology.

[14] Greiner J.V., Oliver P.J., Baker T.L., Lindsay M.E., McPherson A.T., Bedarf G.E., Glonek T. (2022). A recalcitrant skin fissure treated with an anionic polar phospholipid emulsion. Fam. Med. Prim. Care Open Access.

[15] Tan G., Xu P., Lawson L.B., He J., Freytag L.C., Clements J.D., John V.T. (2010). Hydration effects on skin microstructure as probed by high-resolution cryo-scannning electron microscopy and mechanistic implications to enhanced transcutaneous delivery of biomacromolecules. J. Pharm. Sci..

[16] Larsson R. (1994). Lipids-Molecular Organization. Physical Functions and Technical Applications.

[17] Rawlings A.V., Hamilton R.J. (1995). Skin waxes: Their composition, properties, structures and biological significance. Waxes: Chemistry, Molecular Biology and Functions.

[18] Miller W., Bose H. (2011). Early steps in steroidogenesis: Intracellular cholesterol trafficking. J. Lipid Res..

[19] Leszczynski D.E., Schafer R.M. (1990). Nonspecific and metabolic interactions between steroid hormones and human plasma lipoproteins. Lipids.

[20] Scanu A. (1978). Ultrastructure of Serum High Density Lipoproteins: Facts and Models. Lipids.

[21] Kontush A., Lhomme M., Chapman M. (2013). Unraveling the complexities of the HDL lipidome. J. Lipid Res..

